# Giant left coronary artery diagonal branch left ventricular fistula: A case report and review of literature

**DOI:** 10.3389/fcvm.2022.978154

**Published:** 2022-09-06

**Authors:** Jingyue Wang, Huicong Zhang, Qian Tong, Quanwei Wang

**Affiliations:** Department of Cardiology, The First Hospital of Jilin University, Changchun, China

**Keywords:** congenital cardiovascular disease, coronary artery fistula, diagnosis, review, case

## Abstract

A 37-year-old Chinese man was admitted to the department of cardiology of the First Hospital of Jilin University for intermittent palpitation for 9 months, aggravating with chest pain for 3 days. After several examinations, he was diagnosed with giant left ventricular fistula of the diagonal branch of the left coronary artery. After routine treatment, which included improving circulation and administration of dual antiplatelet as well as hypolipidemic drugs among others, the patient’s symptoms did not improve. The fistula was too big for transcatheter occlusion to be performed. A multi-disciplinary suggestion was that the patient be subjected to “surgical closure treatment”; however, for personal reasons, he refused the operation. After discharge, oral beta-blockers were prescribed for the patient. Incidences of congenital coronary arterial fistula in congenital cardiovascular disease are rare, and incidences of the giant fistula being located in the left heart system are even rarer. We report an adult male with a giant left anterior descending diagonal coronary artery left ventricular fistula and show various accessory examination results. Non-invasive ultrasonic cardiography was the first diagnostic option for the disease and pre-admission evaluation. Auxiliary diagnosis and exclusion value of cardiovascular magnetic resonance (CMR) were revealed for the first time. Invasive coronary angiography (ICA) was demonstrated to be the gold standard method again and it was also found that computed tomography angiography (CTA) might be used instead of ICA for determining the exact relationships among anatomic structures. Furthermore, we performed a literature review on the diagnosis and treatment of patients with this condition.

## Introduction

Coronary artery fistula (CAF) refers to an abnormal coronary artery that bypasses the myocardial capillary network and terminates into any cardiac lumen or large vessel. It is characterized depending on the number, origin, course, termination, and presence of an aneurysm or stenotic lesion (1). It is a very rare coronary artery anomaly whose prevalence in the general population is estimated to be 0.002% (2). Even very small CAFs in children require close attention as they may develop with age (3). Our case was an adult male with intermittent palpitation and chest pains due to the left coronary artery diagonal branch left ventricular fistula.

## Case report

**FIGURE 1:**
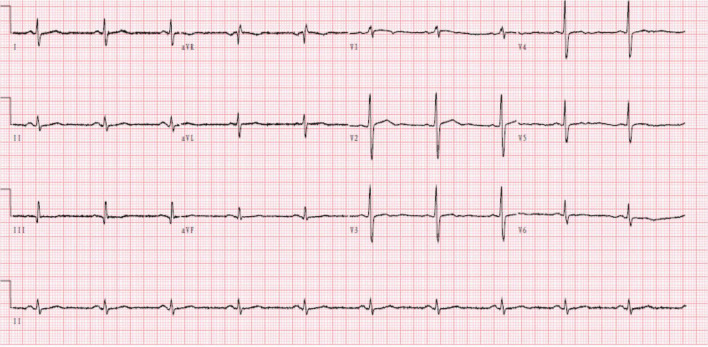
Electrocardiogram: normal, sinus rhythm.

**FIGURE 2:**
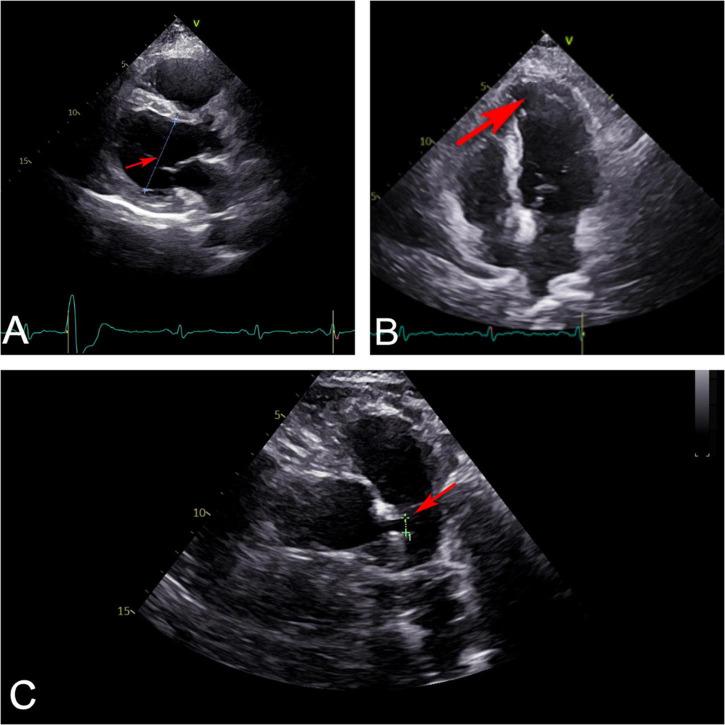
Ultrasonic cardiography showed that **(A)** left ventricle (red arrow) slightly enlarged from the parasternal long axis section view. **(B)** Apex of left ventricle (red arrow) bulged slightly outward from four-chamber view. **(C)** Left main coronary artery (red arrow) widened from random view.

**FIGURE 3:**
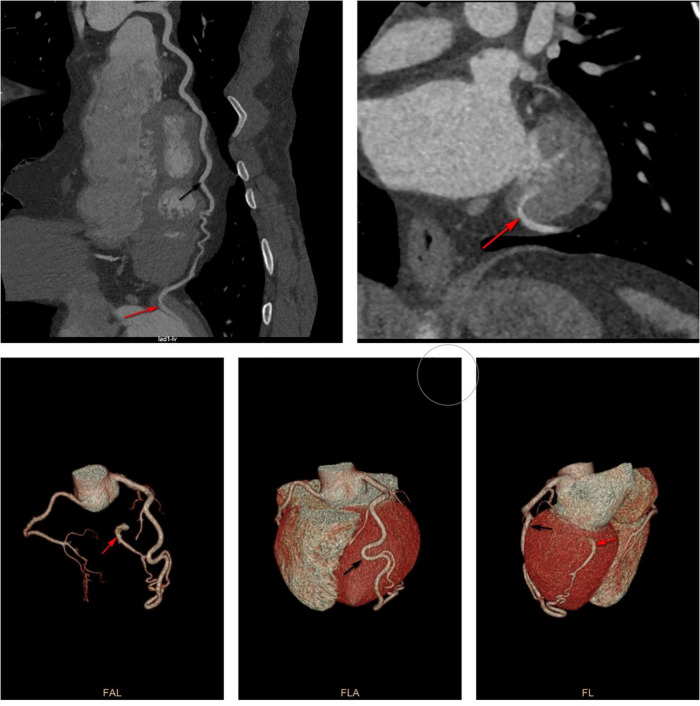
Computed tomography angiography showed that the diagonal branch of the left coronary artery was twisted, lengthened, expanded, extended along the left heart margin, and its distal end penetrated the myocardium from the basal segment of the left ventricular posterior edge into the left ventricle. Black arrow shows the thick diagonal branch; red arrow shows coronary artery-left ventricular fistula.

A 37-year-old Chinese man presenting with untreated palpitation, nausea, and fatigue for 9 months and with worsening palpitation symptoms accompanied by precordial pain was admitted to our hospital. The pain radiated to both shoulders, lasted about 30 min, and improved by itself. The patient used to be physically healthy and had no family history of genetically related diseases, history of trauma and surgery, and no record of drinking. He had a history of smoking for more than 10 years, 1 pack a day, which he never quit until hospitalization. Physical examination revealed: temperature, 36.2°C; pulse, 93 beats/min; breathing, 18 times/min, and blood pressure, 132/78 mmHg. The rest of physical examination did not show any obvious abnormalities. The primary laboratory data are shown in Table 1 according to the time line of the patient’s admission. The patient’s electrocardiogram on admission was normal (Figure 1). Ultrasonic cardiography (UCG) showed left ventricular ectasia (Figure 2). The patient underwent computed tomography angiography (CTA) examination, which suggested a diagonal branch of coronary artery-left ventricular fistula (Figure 3), and was admitted to the cardiology department.

**TABLE 1:** The patient’s laboratory data according to the time line of the admission.

Parameter	Value	References value	Unit	Time
Creatine kinase isoenzyme	1.20	0–4.3	ng/mL	DAY1
Myoglobin	88.40	0–107	ng/mL	DAY1
D-dimer	<100	100–600	ng/mL	DAY1
B-type natriuretic peptide	<5	0–100	ng/mL	DAY1
Troponin	<0.05	0–0.05	ng/mL	DAY1
Creatinine	78.9	57–97	umol/L	DAY1
Urea	7.09	3.1–8.0	mmol/L	DAY1
Serum potassium	3.66	3.5–5.3	mmol/L	DAY1
White blood cell	9.59	3.50–9.50	10^9/L	DAY1
Absolute neutrophil count	6.11	1.80–6.30	10^9/L	DAY1
Hemoglobin	186	130–175	g/L	DAY1
Platelet	243	125–350	10^9/L	DAY1
Activated partial thromboplastin time	25.1	21–33	s	DAY1
Urinary protein	1 +	negative	–	DAY2
Urine ketone	1 +	negative	–	DAY2
Urine specific gravity	1.033	1.010–1.025	–	DAY2
Fecal occult blood	negative	negative	–	DAY2
Aspartate aminotransferase	24.5	15.0–40.0	U/L	DAY2
Alanine transaminase	27.1	9.0–50.0	U/L	DAY2
Albumin	45.1	40.0–55.0	g/L	DAY2
Uric acid	407	210–430	umol/L	DAY2
Cholesterol	5.71	2.6–6.0	mmol/L	DAY2
Triacylglycerol	1.04	0.28–1.80	mmol/L	DAY2
High-density lipoprotein cholesterol	0.97	0.76–2.1	mmol/L	DAY2
Low-density lipoprotein cholesterol	3.83	Low risk-target value <4.14 Medium risk-target value <3.37 High risk-target value <2.59 Extremely high risk-target value < 2.07	mmol/L	DAY2
Fasting blood glucose	5.30	3.9–6.1	mmol/L	DAY2
Thyroid stimulating hormone	1.344	0.35–4.94	uIU/mL	DAY2
Free triiodothyronine	4.27	2.43–6.01	pmol/L	DAY2
Free thyroxine	16.23	9.01–19.05	pmol/L	DAY2
Immunoglobulin quantitation-IgE	<17.10	<100.00	IU/mL	DAY5

**FIGURE 4:**
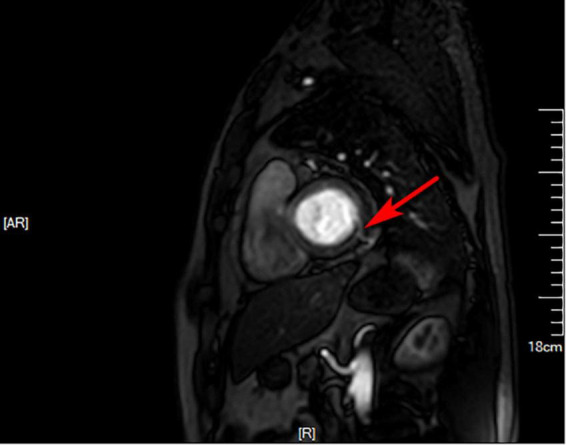
Cardiovascular magnetic resonance. Suspicious fistula at the base of the inferior lateral wall (red arrow) was seen from the left ventricular short axis at 4 o’clock direction.

**FIGURE 5:**
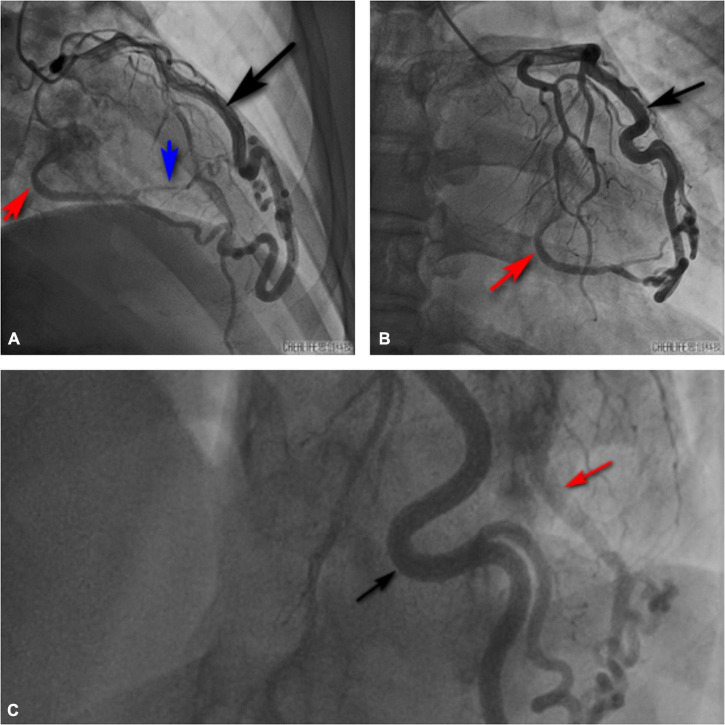
Invasive coronary angiography also showed that the diagonal branch of the left coronary artery was twisted, lengthened, expanded, extended along the left heart margin, and its distal end penetrated the myocardium from the basal segment of the left ventricular posterior edge into the left ventricle. Black arrow shows the thick diagonal branch; red arrow shows the coronary artery-left ventricular fistula; blue arrow shows the branch of coronary artery fistula that supplies the left ventricular posterior wall myocardium. **(A)** Right cranial view. **(B)** Anteroposterior view. **(C)** Left cranial view.

After admission, cardiovascular magnetic resonance (CMR) imaging and invasive coronary angiography (ICA) were performed. CMR of the heart showed suspicious fistula at the base of the inferior lateral wall (Figure 4) while ICA showed similar findings as CTA (Figure 5).

Treatment plans were: after admission, the patient was treated with papaverine, 120 mg, one time a day (QD), intravenous (I.V.); nicotinamide, 400 mg, QD, I.V.; shensongyangxin, 0.8 g, three times a day (TID), by mouth (P.O.); atorvastatin, 20 mg, QD, P.O.; aspirin, 100 mg, QD, P.O. and clopidogrel, 75 mg, QD, P.O. After treatment, there was no obvious improvement in patients’ symptoms. A multi-disciplinary team suggested “surgical closure treatment” under general anesthesia. However, for personal reasons, he refused the operation. After discharge, the patient was prescribed oral beta-blockers.

## Discussion and literature review

Clinically, CAF is a rare cardiac abnormality that should always be considered during differential diagnosis of chest pain and dyspnea, particularly in patients without significant risk factors for acquired heart disease. The etiologies and pathophysiological mechanism of CAF have not been fully established. However, it has been hypothesized that when there is no closure between the trabeculae connecting the coronary arteries, veins, and ventricles, a persistent sinus trabeculation may develop into CAF. As the flow increases, there is a significant increase in coronary branches proximal to the shunt site (4). Due to its hemodynamic consequences or complications, it is associated with various symptoms (5).

In the proximal segments of coronary arteries, CAFs are more likely to form aneurysms, which shows the significance of early diagnosis as early treatment can prevent rupture (3). The diagnostic methods, their advantages, and disadvantages in CAF are summarized in Table 2. Various non-invasive techniques, such as CTA, play a vital role in the diagnosis of these vascular anomalies. The CTA approach is excellent at revealing the origin, course, size, and termination site of CAF as well as its relationship with adjacent anatomic structures (6). We showed non-invasive UCG as the first diagnostic tool and the pre-admission evaluation value for the disease. The auxiliary diagnostic and exclusion values of CMR were assessed for the first time, and the gold standard value of ICA for determining the origin and course of coronary fistula was proven. In this study, CTA showed similar results to ICA. ICA is the commonly used tool, but it is invasive. CTA might be an alternative method for determining the exact relationships among anatomic structures, because of its excellent spatial resolution.

**TABLE 2:** The main diagnostic methods of CAF.

Main diagnostic methods of CAF	Advantages	Disadvantages
Ultrasonic cardiography (UCG)	•Showing abnormal vascular communication in the coronary arteries (6). •Non-invasive. •Measuring shunt flow in selected patients with CAF (7). •Providing excellent qualitative and quantitative assessment of proximal coronary arteries (8).	•Dependending on the operator’s skill (6). •The quality of the acoustic window is poor and the quality of imaging is often limited (6). •Cannot determine whether a coronary fistula is flowing from the posterior atrioventricular sulcus into the right atrium or right ventricle (8).
Transthoracic echocardiography (TTE)	•It has an important complementary role to ICA in depicting the proximal course and flow pattern of abnormal coronary arteries (9). •Useful in accurately depicting the origin, proximal course and flow pattern of anomalous coronary arteries (9). •Helping to determine the precise site of drainage of CAF (10). •The effectiveness of intraoperative TEE in guiding the surgical closure of CAF (10).	•Not indicated in overweight patients (11).
Computed tomography angiography (CTA)	•Negative results could rule out coronary artery disease (12). •Non-invasive (12). •Identification of anomalous origin and course of coronary arteries, assessment of fistula complexity, and preoperative evaluation (13, 14). •Defining the relationship between the details of the coronary vessels and the mediastinal structures (2).	•Renal insufficiency caution. •Contraindication of contrast agent allergy. •Poor image quality due to lower spatial and temporal resolution, motion and blooming artifacts, and adequate image acquisition (15). •Depending on a low and stable heart rate (15). •The amount of radiation (2).
Cardiovascular magnetic resonance (CMR)	•In addition to assessing the anatomy of the fistula, it is possible to further measure the blood flow in its lumen (16). •To provide accurate measurements of cardiac output, shunt flow, turbulent floating jet areas, and even regurgitation (16). •Velocity phase contrast images of the transverse aortic plane can provide the most accurate measurements of cardiac output, shunt, aortic or pulmonary regurgitation, and indirect mitral regurgitation (16).	•Regurgitant valves or severely stenosed aortic valves, which may fragment and are not suitable for accurate velocity measurements by CMR (16).
Multidetector computed tomography (MDCT)	•Acquisition of abnormalities in the aorta, pulmonary arteries, other vascular structures, and cardiac chambers (17). •High temporal and spatial resolution without additional radiation exposure and contrast agents, and the ability to assess the precise anatomical relationship of coronary-pulmonary artery fistulas (17). •Non-invasive (18). Much faster than CMR and can be done in a single breath hold (2). •Higher temporal and spatial resolution than MR imaging (2). •Providing an excellent overview of cardiac and vascular anatomy and helping surgeons understand the complexity of the anatomy prior to surgery (13).	•The amount of radiation (2). •The inability to directly measure pressure in the blood vessels or ventricles is a limitation of all imaging modalities.
Invasive coronary angiography (ICA)	•Outlining the proximal course of the involved coronary artery and fistula (2). •Remains the gold standard for describing the anatomy and collateral circulation of the involved coronary artery, the course of the fistula, the lumen of the receiving heart, and the exact site of communication (19).	•If it is a low-pressure room, it may not show up well (2). •It is usually not possible to adequately fill an aneurysmal CAF with contrast, and it is challenging to clarify the distal site of the CAF and the relationship between the CAF and other cardiac structures (17). •Invasive techniques that require patients to be hospitalized (20). •Only the intraluminal route of the lesion is shown and may prevent a full assessment due to the overlap between tortuous fistulas and adjacent cardiovascular structures (21).

When considering clinical treatment indications and options for CAF, an accurate assessment of the clinical presentation and morphology, including anatomic origin and course, drainage site, as well as possible aneurysm is necessary (1). The American College of Cardiology and American Heart Association guidelines for managing congenital heart disease (CHD) in adults (2008) emphasizes that large CAFs should be closed after their course has been determined, regardless of symptoms (Class I, Level of Evidence: C); small or medium-sized fistulas should be closed if the patient presents with symptoms such as myocardial ischemia, arrhythmias, ventricular dilatation, or dysfunction of unknown origin, or if the fistula is complicated with endocarditis (Class I, Level of Evidence: C); Patients with small asymptomatic fistulas should not be treated but managed by clinical follow-up, including UCG every 3–5 years (Class III, Level of Evidence: C) (22). Symptomatic patients and those with large diameter CAF, whether symptomatic or not, should have their fistulas closed surgically or with transcatheter closure (5). There is consensus regarding the surgical treatment of patients with symptomatic CAF (23). Intracardiac surgical closure of CAF is appropriate for patients with late-onset, large fistulas, coronary arteries with aneurysms, and those who are not candidates for transcatheter treatment (23). Surgical or transcatheter treatments are linked to many risks and operative complications, such as procedural ST-T changes, and postoperative fever (5). Long-term follow-up is required to assess the effectiveness of management, recurrence, and late outcomes (23). Untreated large fistulas might lead to congestive heart failure and premature coronary arterial disease in affected vessels (24). There is no consensus regarding treating asymptomatic adult patients without significant shunts to prevent fistula-related complications (1). Although most patients with such anomalies are asymptomatic, early treatment is recommended to prevent the onset of complications, such as ventricular wall tumor, heart valve disease, cardiomyopathy, and infective endocarditis (25). Armsby et al. (26) performed transcatheter occlusion in 33 of 39 asymptomatic patients with a typical murmur and reported that all patients who accepted interventional therapy had good long-term prognostic outcomes. Researchers are still investigating suitable drugs for the disease. According to Karazisi et al. (5), antiplatelet or warfarin therapies should be considered, especially in coronary artery dilatation. For patients subjected to interventional operation, anticoagulation should be administered after operation. There are various drugs for different symptoms, such as drugs (beta-blockers or calcium channel blockers) for angina and those for treating high-risk factors (hyperlipidemia, hypertension, and diabetes among others). However, these recommendations are mostly empiric (5). Lifelong follow-up is always necessary to ensure that patients with CAF have no disease progression or further cardiac complications. In addition, the risk of infective endocarditis in those patients is also higher than that of ordinary people (11). At present, the patient demanded for conservative treatment and was informed of the above risk. He was also advised to receive regular UCG examination. Most cases of CAF are congenital. CHD is associated with many genes, such as chromatin modifiers, cilia, cilia transduction cell signaling, and maternal factors (27). Cilia and chromatin modifiers may drive the complex genetics of CHD (27). There are many hypotheses regarding the congenital etiologies of CAF. However, the specific molecular mechanisms underlying CAF pathogenesis have not been fully established. Targeted or causative therapies should be investigated through genomics, particularly the study of genes and receptors.

All of the above-mentioned diagnostic methods and treatment options have their merits and demerits. The best diagnostic and treatment plans should be selected based on patient condition and hospital facilities. UCG could be used for preliminary disease screening, CTA might be used instead of ICA for determining the exact relationships with anatomic structures, whereas CMR can be used to exclude other diseases hence help in the diagnosis. In terms of treatment plans, studies should aim at assessing various treatments to inform on the accurate treatment of CAF.

## Data availability statement

The original contributions presented in this study are included in the article/supplementary material, further inquiries can be directed to the corresponding author/s.

## Ethics statement

The studies involving human participants were reviewed and approved by the First Hospital of Jilin University Ethics Committee. The ethics committee waived the requirement of written informed consent for participation.

## Author contributions

JW conceived the idea and conceptualized the case. JW and QW collected the data. JW and HZ analyzed the data and drafted the manuscript. QT and QW reviewed the manuscript. All authors contributed to the article and approved the submitted version.
